# Association of major adverse cardiovascular events with the apnea-hypopnea index, desaturation severity parameters, and cardiac troponins in participants of the Akershus Sleep APnea cohort

**DOI:** 10.1093/sleep/zsag013

**Published:** 2026-01-23

**Authors:** Xin Feng, Fjola Sigurdardottir, Erna Sif Arnardottir, Toril Dammen, Gunnar Einvik, Henri Korkalainen, Ole Klungsøyr, Timo Leppänen, Inger Hilde Nordhus, Sami Nikkonen, Juha Töyräs, Thea Thorshov, Tonje Caroline Øverby, Torbjørn Omland, Harald Hrubos-Strøm

**Affiliations:** Department of Research and Innovation, Division of Mental Health and Addiction, Oslo University Hospital, Oslo, Norway; Division of Mental Health and Addiction, Institute of Clinical Medicine, Faculty of Medicine, University of Oslo, Oslo, Norway; Department of Otorhinolaryngology, Division of Surgery, Akershus University Hospital, Lørenskog, Norway; K.G. Jebsen Center for Cardiac Biomarkers, Institute of Clinical Medicine, University of Oslo, Oslo, Norway; Department of Engineering and Department of Computer Science, Reykjavik University, Reykjavik, Iceland; Department of Research and Innovation, Division of Mental Health and Addiction, Oslo University Hospital, Oslo, Norway; Division of Mental Health and Addiction, Institute of Clinical Medicine, Faculty of Medicine, University of Oslo, Oslo, Norway; Pulmonary Department, Division of Medicine, Akershus University Hospital, Lørenskog, Norway; Division of Medicine and Laboratory, Institute of Clinical Medicine, Faculty of Medicine, University of Oslo, Oslo, Norway; Department of Technical Physics, University of Eastern Finland, Kuopio, Finland; Diagnostic Imaging Center, Kuopio University Hospital, Kuopio, Finland; Department of Research and Innovation, Division of Mental Health and Addiction, Oslo University Hospital, Oslo, Norway; Division of Mental Health and Addiction, Institute of Clinical Medicine, Faculty of Medicine, University of Oslo, Oslo, Norway; Department of Technical Physics, University of Eastern Finland, Kuopio, Finland; Diagnostic Imaging Center, Kuopio University Hospital, Kuopio, Finland; School of Electrical Engineering and Computer Science, The University of Queensland, Brisbane, Australia; Department of Clinical and Biological Psychology, Faculty of Psychology, University of Bergen, Bergen, Norway; Department of Technical Physics, University of Eastern Finland, Kuopio, Finland; Diagnostic Imaging Center, Kuopio University Hospital, Kuopio, Finland; Department of Technical Physics, University of Eastern Finland, Kuopio, Finland; School of Electrical Engineering and Computer Science, The University of Queensland, Brisbane, Australia; Science Service Center, Kuopio University Hospital, Kuopio, Finland; Department of Otorhinolaryngology, Division of Surgery, Akershus University Hospital, Lørenskog, Norway; Division of Surgery, Institute of Clinical Medicine, Faculty of Medicine, University of Oslo, Oslo, Norway; Department of Otorhinolaryngology, Division of Surgery, Akershus University Hospital, Lørenskog, Norway; Division of Surgery, Institute of Clinical Medicine, Faculty of Medicine, University of Oslo, Oslo, Norway; K.G. Jebsen Center for Cardiac Biomarkers, Institute of Clinical Medicine, University of Oslo, Oslo, Norway; Department of Cardiology, Division of Medicine, Akershus University Hospital, Lørenskog, Norway; Department of Otorhinolaryngology, Division of Surgery, Akershus University Hospital, Lørenskog, Norway; Division of Surgery, Institute of Clinical Medicine, Faculty of Medicine, University of Oslo, Oslo, Norway

**Keywords:** obstructive sleep apnea, cardiovascular disease, polysomnography, oxygen desaturation parameters, troponin

## Abstract

**Study Objectives:**

To examine the prognostic value of the apnea-hypopnea index (AHI), desaturation severity parameters, and cardiac troponins alone and combined for major cardiovascular events (MACEs).

**Methods:**

MACE data were retrieved in 2021 from the Norwegian Patient Registry for 518 participants in the Akershus Sleep APnea (ASAP) cohort. Baseline polysomnography and fasting blood samples were collected between June 2006 and January 2008. Desaturation duration (DesDur) and severity (DesSev) were calculated using Automatic Blood Oxygen Saturation Analysis software. Cox regression models estimated hazard ratios (HRs) for MACE. Predictive properties of combining troponins and obstructive sleep apnea severity were calculated by comparing established clinical thresholds for cardiac troponin I (cTnI) and T (cTnT) with AHI clinical thresholds of ≥15 and ≥30, respectively.

**Results:**

High AHI, DesDur, DesSev, cTnI, and cTnT were associated with increased MACE risk. However, only cTnI independently predicted MACE after adjustment (HR: 1.74, 95% CI: 1.32–2.29). The HR for MACE was 2.68 (95% CI: 1.03–6.97) in patients with both high cTnI and AHI ≥30 events/h.

**Conclusion:**

In this 15-year follow-up, cTnI was independently associated with risk of MACE, whereas the AHI, desaturation parameters, and cTnT were not independent predictors. cTnI, especially when combined with AHI, was a stronger MACE predictor than cTnT. Provided our findings are validated in clinical obstructive sleep apnea populations, the measurement of cTnI may be considered for cardiovascular risk stratification.

## Teaser

**Statement of Significance** This research provides important insights into the prediction of major adverse cardiovascular events (MACEs) in participants of a community-based cohort defined by high and low risk of obstructive sleep apnea (OSA). Risk stratification based on OSA severity, followed by a cardiac troponin I test, resulted in more accurate assessment of MACE risk than using either marker alone. Notably, validation in clinical OSA cohort is needed before the integration of multiple biomarkers for MACE prediction can be recommended.

## Graphical Abstract

**Graphical Abstract:**
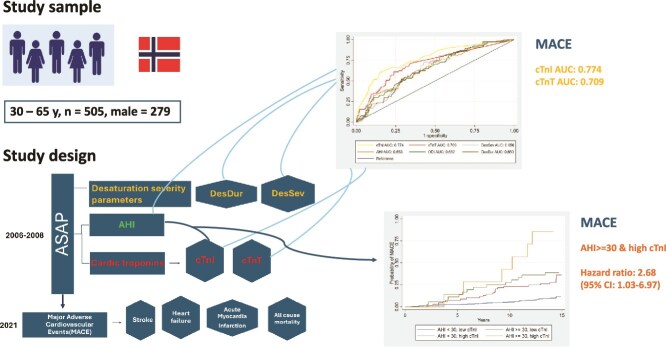

## Introduction

Increased risk of major cardiovascular events (MACEs) in obstructive sleep apnea (OSA) patients has been reported previously [1–5]. However, there is ongoing debate on how to best utilize information from polysomnographic (PSG) recordings for risk prediction [6, 7]. The apnea-hypopnea index (AHI) and oxygen saturation measures calculated from PSG were introduced nearly five decades ago to diagnose and estimate the severity of OSA [8]. Despite this, these metrics are poorly correlated with OSA-related symptoms and outcomes, and data on their association with the incidence of MACE are inconsistent [9].

Novel oxygen saturation (SpO2)-based parameters, such as desaturation duration (DesDur) and desaturation severity (DesSev) [10], have been proposed for more accurate MACE prediction [10, 11]. Much later, Azarbazin and colleagues coined the term “hypoxic burden,” and demonstrated that it also is a better risk predictor for MACE than AHI [3, 12]. The OSA-specific hypoxic burden has been applied as a parameter to demonstrate the differences between intervention and control groups in a prospective study [13]. Both DesSev [10] and hypoxic burden [3] are capturing the area under the desaturation curve despite slightly differing calculation methods.

In non-OSA populations, cardiac troponins are strong predictors of mortality and cardiovascular disease (CVD) [14]. Specifically, cardiac troponin I (cTnI) and T (cTnT) are both sensitive and specific markers of increased cardiovascular risk [15]. Our previous cross-sectional studies found that higher AHI, higher percentage of total sleep time with SpO2 < 90 per cent (t90), higher DesDur, and DesSev, as well as lower mean SpO2 are independently associated with higher cTnI levels in participants of the Akershus Sleep APnea (ASAP) cohort [16, 17].

To the best of our knowledge, only one large cohort study has reported sex differences in the predictive properties of troponins and biomarkers derived from PSG [18]. Roca et al. found that AHI is significantly associated with incident heart failure and death in women but not in men with OSA; however, this association was no longer significant when cTnT levels were considered. Additionally, the predictive properties of combining these biomarkers were not reported.

The clinical interpretation of the relationships between AHI, desaturation severity parameters, and cardiac troponins in predicting CVD is complex. Therefore, the combined use of these biomarkers may improve clinical precision. We hypothesize that the predictive value of cardiac troponins could be enhanced when combined with clinically established OSA severity stratification. To test our hypothesis, we used data from the ASAP epidemiological cohort to investigate proportional hazards and predictive properties of the AHI and cardiac troponins on MACE. In addition, we aim to study the proportional hazard of the desaturation severity parameters on MACE.

## Materials and Methods

### Study sample

Between June 2006 and January 2008, 518 community-dwelling individuals aged 30 to 65 years participated in the baseline ASAP examination. Of these participants, 365 were assessed as high risk and 153 as low risk for OSA using the Berlin Questionnaire (BQ) [19]. All participants provided written, informed consent to participate in the study.

### Baseline examinations

Participants underwent a comprehensive clinical examination, including standardized measurements of height, weight, and blood pressure, as well as an in-hospital PSG using the Embla system (Flaga Medical, Reykjavik, Iceland). The in-lab PSG data were analyzed with Somnologica 3.2 software (Flaga-Medcare, Buffalo, NY, USA) by two experienced US board-certified sleep technicians. An apnea was scored if airflow dropped by ≥90 per cent from the reference amplitude for more than 10 s. A hypopnea was scored when airflow dropped by ≥30 per cent from the reference amplitude for more than 10 s and was followed by a subsequent oxygen desaturation of ≥4 per cent. The AHI was calculated as the average number of apneas and hypopneas per hour of sleep [20]. More detailed scoring and technical information have been reported previously [21].

Additionally, two novel parameters representing the morphology and duration of oxygen desaturations, DesDur and DesSev, were calculated using an open-source software Automatic Blood Oxygen Saturation Analysis (ABOSA) [22]. European Data Format files were uploaded to the software and desaturation events automatically scored after selecting the minimum 5 s duration and 4 per cent minimum desaturation drop as criteria. DesDur is the sum of the durations of all desaturation events divided by total sleep time, thus reflecting the duration of desaturation events. In addition, DesSev is the sum of the areas of all desaturation events normalized by total sleep time and irrespective of respiratory events, therefore containing information on both the depth and duration of desaturation. More detailed technical information on scoring and formulas for the parameters have been published previously [23].

Laboratory measurements were obtained from fasting venous whole blood samples collected in the morning after the in-hospital PSG. Blood sampling and processing were conducted by dedicated study personnel at the Akershus University Hospital. cTnI and cTnT were analyzed using the ARCHITECT STAT High-Sensitivity Troponin assay (Abbott Diagnostics, Abbott Park, IL, USA) on a Cobas e411 platform (Roche Diagnostics, Rotkreuz, Switzerland). Detailed descriptions of the troponin measurements have been provided previously [24].

Co-morbid CVDs, including heart failure, heart valve disease, coronary artery bypass grafting, percutaneous coronary intervention, atrial fibrillation, arrhythmia, stroke, and other cerebrovascular diseases, were recorded during a structured baseline interview supported by access to the electronic patient journal. Cardiovascular risk factors such as hypertension, diabetes, and smoking were recorded during the interview. The glomerular filtration rate was calculated [25], and creatinine, fasting glucose, and blood lipids were measured using standard laboratory methods [26].

### Outcomes

A cardiovascular event was defined according to the International Classification of Diseases (ICD-10) [27] and identified through the Norwegian Patient Registry. Deaths were confirmed using patients’ medical records from hospital records. The MACE outcome included time to first incident all-cause mortality, acute myocardial infarction, heart failure, and stroke during the follow-up period from January 1, 2008, to April 30, 2021. Censoring was determined at the date of all-cause mortality, acute myocardial infarction, heart failure, stroke, or the end of follow-up, whichever occurred first.

### Ethics

The study protocol was approved by the Regional Committee for Medical Research Ethics in eastern Norway (ID 13843), and the hospital personal security officer in 2005.

### Statistical analyses

Continuous variables are presented as mean ± standard deviation (*SD*), and categorical variables are presented as absolute numbers and percentages. cTnI and cTnT were logarithmically transformed before analysis, as previously reported [17]. For further analyses, we divided the participants into MACE and non-MACE groups.

To investigate the association between MACE and different prespecified biomarkers (i.e. AHI, ODI, DesDur, DesSev, cTnI, and cTnT), we used Cox proportional hazard models adjusted for putative confounders to study the relationships between predictive variables and MACE. Four different Cox models were utilized: model 1, unadjusted; model 2, adjusted for age and sex; model 3, adjusted for age, sex, body mass index (BMI), smoking status, diabetes, hypertension, low density lipoprotein cholesterol (LDL-C) and CVD and model 4, adjusted for age, sex, BMI, smoking status, diabetes, hypertension, LDL-C, CVD, and Epworth Sleepiness Scale (ESS). Additionally, receiver operating characteristic curves were plotted and areas under the curves (AUC) were calculated to compare prognostic merit for MACE of different predictor variables.

As the severity of OSA is often divided into mild, moderate, and severe categories by well-established AHI cut-off values [28], participants were divided into two dichotomized groups for the analysis of predictive properties: (1) moderate-to-severe OSA (AHI ≥ 15 events/h) versus no-to-mild OSA (AHI < 15 events/h) and (2) severe OSA (AHI ≥ 30 events/h) versus non-severe OSA (AHI < 30 events/h). Furthermore, cTnI and cTnT were dichotomized into low and high based on established clinical cut-off values: for cTnI, 4 ng/L for women and 6 ng/L for men [29], and for cTnT, 7 ng/L for women and 8 ng/L for men [30].

Dichotomized AHI and cTnI/cTnT were combined to stratify groups for Cox proportional regression models to calculate HRs for MACE. To investigate the additional prognostic value of troponins across different OSA severity strata, the following reference groups were used: (1) Participants with AHI ≥ 15 and low cTnI/cTnT; (2) Participants with AHI ≥ 30 events/h and low cTnI/cTnT. Complement of Kaplan–Meier curves were utilized to illustrate the unadjusted time to MACE in the stratified groups.

Statistical analyses were performed using Stata/SE 16.1 (StataCorp LLC, College Station, TX). A *p*-value of .05 was set as the threshold for statistical significance.

## Results

Among the 518 participants, four were excluded due to missing AHI or SpO2 data. Additionally, data on cTnI and/or cTnT were missing for nine participants. Therefore, 505 participants were available for analysis. The baseline characteristics for the MACE and non-MACE groups are shown in Table 1.

**Table 1:** Comparison of Akershus Sleep Apnea project participants’ baseline characteristics between major adverse cardiovascular events (MACE) and Non-MACE groups.

Total (*n* = 505)	MACE (*n* = 81)	Non-MACE (*n* = 424)
Age (years), mean (*SD*)	57.1 (7.4)	46.8 (11.0)
Male sex, *n* (%)	64 (79.0)	215 (50.7)
Current smoker, *n* (%)	23 (28.3)	110 (25.9)
CVD, *n* (%)	33 (40.7)	50 (11.7)
Hypertension, *n* (%)	71 (87.6)	213 (50.2)
LDL-C^*^, *n* (%)	52 (65.0)	311 (74.9)
Diabetes mellitus, *n* (%)	24 (29.6)	38 (8.9)
eGFR (mL/min), mean (*SD*)	115.6 (29.6)	126.4 (37.8)
SBP (mmHg), mean (*SD*)	142.4 (17.6)	134.0 (17.8)
BMI (kg/m^2^), mean (*SD*)	29.9 (4.9)	28.7 (5.0)
ESS^†^, mean (*SD*)	8.2 (4.4)	8.7 (4.2)
Polysomnographic parameters		
Mean SpO_2_, %, mean (*SD*)	93.2 (1.9)	94.7 (1.7)
Min SpO_2_, %, mean (*SD*)	82.6 (6.2)	85.6 (6.4)
t90 (min), mean (*SD*)	27.1 (44.3)	10.7 (27.8)
TST (h), mean (*SD*)	6.5 (1.1)	7.1 (1.3)
AHI (events/h), mean (*SD*)	21.2 (19.4)	12.6 (17.7)
ODI (events/h), mean (*SD*)	20.9 (20.1)	11.9 (16.2)
DesDur^‡^ (%-point), mean (*SD*)	15.8 (14.3)	10.6 (12.3)
DesSev^‡^ (%-point), mean (*SD*)	0.63 (0.90)	0.39 (0.62)
Circulating cardiac biomarkers		
cTnI, mean (*SD*)	7.3 (15.6)	2.2 (2.3)
cTnT, mean (*SD*)	8.0 (6.3)	4.8 (3.7)

Abbreviations: AHI, apnea-hypopnea index; BMI, body mass index; CVD, cardiovascular disease; cTnI, cardiac troponin I; cTnT, cardiac troponin T; DesDur, desaturation duration; DesSev, desaturation severity; eGFR, estimated glomerular filtration rate; ESS, Epworth Sleepiness Scale; IQR. inter quartile range; LDL-C, low density lipoprotein cholesterol; ODI, oxygen desaturation index; SBP, systolic blood pressure; SpO_2_, lowest oxygen saturation; *SD*, standard deviation; t90, total sleep time with oxygen saturation < 90%; TST, total sleep time.

^*^The cut-off value was set at LDL-C ≥ 3 mmol/L, the dataset had 10 missing values (MACE, *n* = 80).

^†^Six missing values (MACE, *n* = 80).

^‡^DesDur and DesSev were calculated for 458 participants (MACE, *n* = 77).

All continuous predicting variables examined (AHI, ODI, DesDur, DesSev, cTnI, and cTnT) were significantly associated with MACE in the unadjusted Cox regression models (Table 2). However, the proportional hazards ratios for AHI, ODI, DesDur, and DesSev were reduced and did not show significant HRs after adjustment for age and sex. cTnI and cTnT categories independently predicted MACE, with hazard ratios (HRs) of 1.81 (95% CI: 1.40–2.35) and 1.53 (95% CI: 1.06–2.19), respectively, after adjusting for age, sex, BMI, smoking status, diabetes, hypertension, LDL-C, and baseline CVD. Only cTnI independently predicted MACE, with HR of 1.74 (95% CI: 1.32–2.29) after adding ESS to the fully adjusted model.

**Table 2:** Cox regression analysis for major adverse cardiovascular events (MACE).

**MACE** Hazard ratio (95% CI)	**Model**	**AHI**	**ODI**	**DesDur** ^*^	**DesSev**	**cTnI**	**cTnT**
	**1**	1.01 (1.00–1.02)^*^^*^^*^	1.02 (1.00–1.02)^*^^*^^*^	1.02 (1.01–1.03)^*^^*^	1.41 (1.13–1.77)^*^^*^	2.44 (2.04–2.94)^*^^*^^*^	2.70 (2.02–3.59)^*^^*^^*^
	**2**	1.00 (0.99–1.01)	1.00 (0.99–1.01)	1.00 (0.98–1.02)	1.12 (0.83–1.49)	1.85 (1.46–2.34)^*^^*^^*^	1.63 (1.14–2.32)^*^^*^
	**3**	0.99 (0.98–1.01)	1.00 (0.99–1.01)	0.99 (0.98–1.01)	1.00 (0.73–1.36)	1.81 (1.40–2.35)^*^^*^^*^	1.53 (1.06–2.19)^*^
	**4**	0.99 (0.98–1.00)	1.00 (0.99–1.00)	0.99 (0.98–1.01)	1.00 (0.73–1.37)	1.74 (1.32–2.29)^*^^*^^*^	1.42 (0.98–2.06)

Model 1: Univariate unadjusted Cox regression; Model 2: Model 1 + adjusted for age and sex; Model 3: Model 2 + adjusted for BMI, smoking status, diabetes, hypertension, LDL-C^†^, and baseline CVD; Model 4: Model 3 + adjusted for ESS. ^*^p < .05, ^**^p < .01, ^***^p < .001.Abbreviations: AHI, apnea-hypopnea index; BMI, body mass index; CI, confidence interval; CVD, cardiovascular disease; cTnI, cardiac troponin I; cTnT, cardiac troponin T; DesDur, desaturation duration; DesSev, desaturation severity; ESS, Epworth Sleepiness Scale; LDL-C, low density lipoprotein cholesterol; MACE, major adverse cardiovascular events; ODI, oxygen desaturation index.

^*^DesDur and DesSev were calculated for 458 participants (MACE, *n* = 77).

^†^The cut-off value was set at LDL-C ≥ 3 mmol/L.

**Figure 1:**
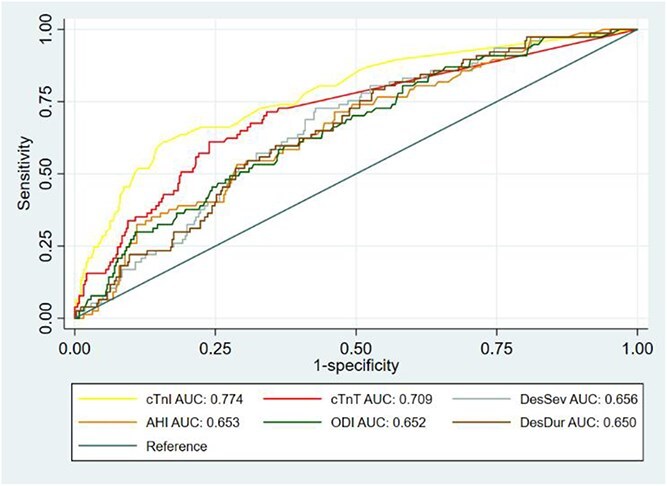
Receiver operating curves (ROC) with area under the curve (AUC) for the comparison of apnea-hypopnea index (AHI), oxygen desaturation index (ODI), desaturation duration (DesDur), desaturation severity (DesSev), cardiac troponin I, and cardiac troponin T for predicting major adverse cardiovascular events, unadjusted (*n* = 458).

In the unadjusted analysis of MACE, the AUC values ranked from highest to lowest are cTnI (0.774), cTnT (0.709), DesSev (0.656), AHI (0.653), ODI (0.652), and DesDur (0.650). (Figure 1). The distributions of predictors stratified by MACE status, were illustrated as box plots in Figure S1.

The prognostic properties of AHI-troponin group combinations are presented in Table 3. Within the moderate-to-severe OSA group (AHI ≥ 15 events/h), high cTnI was associated with higher risk of MACE with a HR of 3.01 (95% CI: 1.41–6.39) in model 1, HR of 2.23 (95% CI: 1.04–4.77) in model 2, and HR of 2.34 (95% CI: 1.07–5.11) in model 3. In the no-to-mild OSA group (AHI < 15 events/h), high cTnI was associated with higher HRs for MACE with an HR of 2.64 (95% CI: 1.31–5.32) in model 1, HR of 3.20 (95% CI: 1.56–6.56) in model 2, and HR of 3.62 (95% CI: 1.73-7.56) in model 3. For cTnT, high cTnT was associated with higher risk of MACE only within the moderate-to-severe OSA group in model 1 (HR = 2.13, 95% CI = 1.09–4.15).

**Table 3:** Hazard ratios for major adverse cardiovascular events (MACE) across categories of the apnea-hypopnea index, cardiac troponin I (cTnI), and cardiac troponin T (cTnT).

MACE/total (*n* = 81/505)		Model 1 Hazard ratio (95% CI)	Model 2 Hazard ratio (95% CI)	Model 3 Hazard ratio (95% CI)	
Stratified groups by the AHI cut-off value of 15 events/h	AHI ≥ 15, low cTnI^*^	1	1	1
	AHI ≥ 15, high cTnI	3.01 (1.41–6.39)^*^^*^	2.23 (1.04–4.77)^*^	2.34 (1.07–5.11)^*^
	AHI < 15, high cTnI	2.64 (1.31–5.32)^*^^*^	3.20 (1.56–6.56)^*^^*^	3.62 (1.73–7.56)^*^^*^
	AHI < 15, low cTnI	0.45 (0.27–0.75)^*^^*^	0.97 (0.57–1.63)	1.14 (0.68–2.02)
	AHI ≥ 15, low cTnT^†^	1	1	1
	AHI ≥ 15, high cTnT	2.13 (1.09–4.15)^*^	1.08 (0.55–2.14)	1.23 (0.61–2.46)
	AHI < 15, high cTnT	1.79 (0.93–3.44)	1.68 (0.87–3.23)	1.91 (0.98–3.72)
	AHI < 15, low cTnT	0.44 (0.25–0.77)^*^^*^	1.08 (0.55–2.14)	1.06 (0.59–1.90)
Stratified groups by the AHI cut-off value of 30 events/h	AHI ≥ 30, low cTnI	1	1	1
	AHI ≥ 30, high cTnI	4.18 (1.67–10.43)^*^^*^	3.35 (1.33–8.38)^*^	2.68 (1.03–6.97)^*^
	AHI < 30, high cTnI	1.46 (0.74–2.89)	1.58 (0.78–3.18)	1.93 (0.94–3.98)
	AHI < 30, low cTnI	0.29 (0.17–0.50)^*^^*^^*^	0.59 (0.34–1.02)	0.70 (0.40–1.24)
	AHI ≥ 30, low cTnT	1	1	1
	AHI ≥ 30, high cTnT	1.82 (0.84–3.93)	1.05 (0.48–2.31)	1.24 (0.56–2.74)
	AHI < 30, high cTnT	1.02 (0.52–2.01)	0.84 (0.42–1.68)	1.08 (0.53–2.17)
	AHI < 30, low cTnT	0.29 (0.16–0.52)^*^^*^^*^	0.53 (0.29–0.97)^*^	0.66 (0.35–1.23)

Abbreviations: AHI = apnea-hypopnea index, BMI = body mass index, CI = confidence interval, CVD = cardiovascular disease, OSA = obstructive sleep apnea. Model 1: Univariate unadjusted Cox regression; Model 2: Model 1 + adjusted for age and sex; Model 3: Model 2 + adjusted for BMI, smoking status, diabetes, hypertension, and baseline CVD. ^*^p < .05, ^**^p < .01, ^***^p < .001.

^*^cTnI = cardiac troponin I: cTnI was considered high if it was ≥4 ng/L for women and ≥ 6 ng/L for men.

^†^cTnT = cardiac troponin T: cTnT was considered high if it was ≥7 ng/L for women and ≥ 8 ng/L for men.

In the severe OSA group (AHI ≥ 30 events/h), high cTnI was associated with higher risk for MACE with an HR of 4.18 (95% CI: 1.67–10.43) in model 1, HR of 3.35 (95% CI: 1.33–8.38) in model 2, and HR of 2.68 (95% CI: 1.03–6.97) in model 3 (Table 3). However, high cTnI was not significantly associated with higher the risk for MACE in the non-severe OSA group (AHI < 30 events/h). Regarding cTnT, only low cTnT in the non-severe OSA group (AHI < 30 events/h) showed a significantly lower risk for MACE, with an HR of 0.29 (95% CI: 0.16–0.52) in model 1 and HR of 0.53 (95% CI: 0.29–0.97) in model 2.

**Figure 2:**
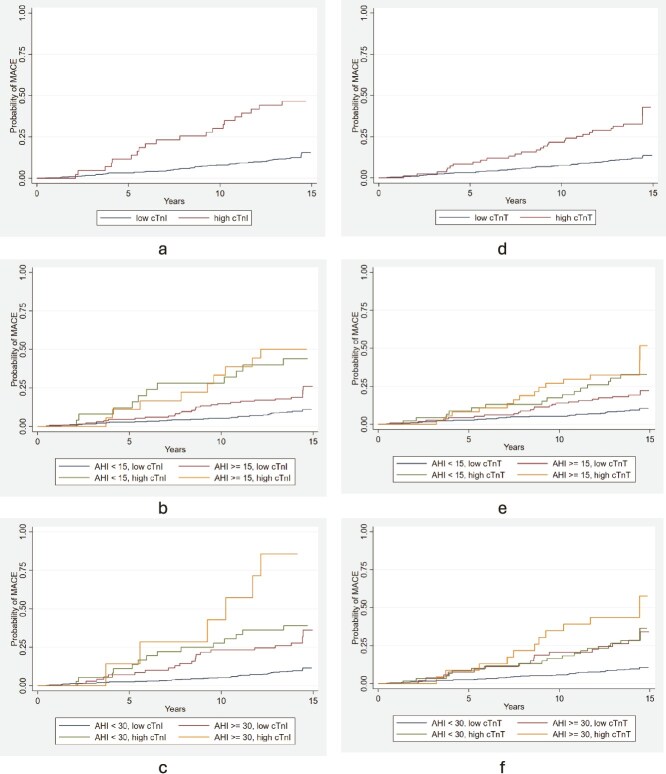
Unadjusted complement of Kaplan–Meier curves for categories of cardiac troponin I (cTnI)/cardiac troponin T (cTnT) and combined with apnea-hypopnea index (AHI), respectively in major adverse cardiovascular events (MACE) in the Akershus sleep Apnea epidemiological cohort. (A) cTnI; (B) AHI < or ≥15 events/h combined with cTnI; (C) AHI < or ≥30 events/h combined with cTnI; (D) cTnT; (E) AHI < or ≥15 events/h combined with cTnT; and (F) AHI < or ≥ 30 events/h combined with cTnT.

Complement of Kaplan–Meier curves demonstrate the unadjusted risks for MACE within different cTnI/cTnT and AHI categories (Figure 2). The combination of AHI ≥ 30 events/h with high cTnI resulted in the highest risk for MACE, followed by the combination of AHI ≥ 30 events/h with high cTnT. Additionally, comparing AHI ≥ 30 events/h with high troponin values showed a higher risk for MACE compared to high cTnI or cTnT alone.

## Discussion

The main finding of this study is that cTnI is a stronger predictor of MACE than AHI, desaturation parameters, and cTnT. Additionally, risk categorization based on combinations of clinically established thresholds for troponins and OSA severity shows promise, particularly for cTnI. None of AHI, DesDur, DesSev, or cTnT did predict MACE after adjustment for potential confounders. The present results confirm our hypothesis that the combined use of AHI and cTnI may enhance risk communication in patients with OSA.

Our finding that troponins are better predictors of MACE than biomarkers derived from PSG aligns with previous findings by Roca et al. [17] However, HRs cannot be directly compared due to the different units and ranges of the variables used. The AHI, ODI, and desaturation parameters do not exhibit significant HRs after adjusting for age and sex, indicating that these parameters may change with increasing age or be influenced by sex. A systematic review reported that the overall prevalence of OSA increases with age, reaching as high as 90 per cent in men and 78 per cent in women in some elderly groups [31]. Our finding that the desaturation severity parameter did not predict MACE contradicts the findings by Karhu et al. [11] and Azarbarzin et al. [12]. However, this discrepancy may be explained by the larger sample size in the Sleep Heart Health Study (SHHS) and different sampling techniques. The participants of the ASAP were stratified by the Berlin Questionnaire from a community-based population, which differs from the SHHS.

Our finding that cTnI and cTnT are significantly associated with the risk of MACE is consistent with previous findings in non-OSA populations [14, 15]. We observed that models incorporating cTnI resulted in higher HRs for MACE compared to models with cTnT (Table 2). These findings align with other population-based studies, which reported that elevated cTnI is independently associated with increased cardiovascular risk [32]. Welsh et al. argued that cTnI, but not cTnT, is associated with myocardial infarction and coronary heart disease in the general population [15]. Conversely, it has been reported that cTnT is more robustly associated with non-CVD death compared to cTnI [15, 33]. These observations suggest that cTnI may have greater cardiac specificity than cTnT, which again may contribute to a stronger association between cTnI and the incidence of MACE. Moreover, we have demonstrated that the AHI, DesSev, and DesDur were associated with cardiac troponin I but not troponin T [17]. In the current analysis, after adjusting for ESS, only cTnI remained significantly associated with MACE, whereas cTnT was no longer a significant predictor. These results suggest that cTnI may be more closely linked to MACE, whereas troponin T may be influenced by other factors such as excessive daytime sleepiness. This distinction highlights the potential differential relevance of cardiac biomarkers in relation to OSA severity and MACE.

In addition to modeling the proportional hazards of AHI, ODI, DesDur, DesSev, and cardiac troponins individually, we found that the predictive properties of cardiac troponins increased when adding clinically established thresholds of AHI ≥ 15 and AHI ≥ 30. Previous cross-sectional studies have shown that severe OSA is associated with higher cTnI levels [16, 34]. Consistent with this, we found that in severe OSA (AHI ≥ 30 events/h), elevated cTnI levels markedly increased the HRs for MACE after adjusting for confounders, suggesting that high cTnI may be a strong predictor of adverse cardiovascular outcomes in individuals with severe OSA. However, AHI ≥ 15 did not differentiate the predictive value for patients with high cTnI. Patients with AHI between 5 and 15 or between 15 and 30 have been reported to exhibit comparable HRs for CVD and all-cause mortality [35]. We emphasize the value of combining AHI ≥ 30 with high cTnI for predicting MACE. Our findings suggest that cTnI levels provide a unique risk stratification opportunity for patients with severe OSA and higher cTnI.

### Strengths and limitations

The main strengths of this study include the baseline assessment of a community-based population using both in-hospital polysomnography and high-sensitivity cardiac troponins, along with a long follow-up period. Additionally, the extensive baseline assessments enabled the adjustment of Cox models for potential confounders.

A limitation of this study was the relatively small sample size, which precluded subgroup analyses. Moreover, a potential selection bias related to the exclusion of respondents with missing values in the Berlin Questionnaire has been reported previously [19]. Additionally, we lack information about the causes of death. By dichotomizing our population (instead of using AHI as a continuous variable), the results for OSA groups with AHI ≥ 30 are not directly comparable to those for OSA patients with AHI ≥ 15, as the reference groups differ. We only combined AHI and troponins, not desaturation severity parameters, with troponins to predict MACE. Future research is needed to determine the optimal cut-off values for these parameters in MACE prediction. As the study relied on registry-based follow-up, potential missing or incorrect entries in the Norwegian Patient Registry may have led to underreporting of MACE events. Additionally, the registry data were available only from 2008; MACEs, except for all-cause mortality that may have occurred between 2006 and 2008, could not be captured, which could result in a slight underestimation of outcomes. A further limitation of this study is that we do not have data available for all the participants for further analyses. Future longitudinal studies with repeated assessments of OSA severity and cardiac troponins are needed to elucidate the temporal and mediating pathways linking OSA to cardiovascular events.

## Conclusion

High-sensitivity cardiac troponin I, but not AHI, desaturation severity parameters, or high-sensitivity cardiac troponin T, was a significant predictor of MACE in participants of the ASAP epidemiological cohort. The combined use of cTnI and AHI predicted MACE better than the combination of cTnT and AHI. Accordingly, a two-step risk evaluation for OSA patients would be beneficial in clinical practice: first, stratify by AHI ≥ 30 events/h, followed by a cTnI test. Combining AHI with cTnI offers a more comprehensive risk stratification, potentially improving early detection and targeted interventions for MACE in OSA patients. These novel findings pave the way for future studies using a stepwise approach to predicting MACE also in clinical populations of OSA patients.

## Supplementary Material

Supplemental_Materials_zsag013

## Data Availability

The data underlying this article will be shared on reasonable request to the corresponding author.
