# Supplementary material for: Association of major adverse cardiovascular events with the apnea-hypopnea index, desaturation severity parameters, and cardiac troponins in participants of the Akershus Sleep APnea cohort
Source: Sleep. 2026 Jan 23;49(8):zsag013. doi: 10.1093/sleep/zsag013 (PMC13472890; doi:10.1093/sleep/zsag013)

Association of major adverse cardiovascular events with the apnea-hypopnea index, desaturation severity parameters, and cardiac troponins in participants of the Akershus Sleep APnea (ASAP) cohort

Xin Feng[1-3], Fjola Sigurdardottir[4], Erna Sif Arnardottir[5], Toril Dammen[1,2], Gunnar Einvik[6,7], Henri Korkalainen[8,9], Ole Klungsøyr[1,2], Timo Leppänen[8-10], Inger Hilde Nordhus[11], Sami Nikkonen[8-9], Juha Töyräs[8,10,12], Thea Thorshov[3,13], Tonje Caroline Øverby[3,13], Torbjørn Omland[4,14], Harald Hrubos- Strøm[3,13]*

1. Department of Research and Innovation, Division of Mental Health and Addiction, Oslo University Hospital, Oslo, Norway

2. Division of Mental Health and Addiction, Institute of Clinical Medicine, Faculty of Medicine, University of Oslo, Oslo, Norway

3. Department of Otorhinolaryngology, Division of Surgery, Akershus University Hospital, Lørenskog, Norway

4. K. G. Jebsen Center for Cardiac Biomarkers, Institute of Clinical Medicine, University of Oslo, Oslo, Norway

5. Department of Engineering and Department of Computer Science, Reykjavik University, Reykjavik, Iceland

6. Pulmonary Department, Division of Medicine, Akershus University Hospital, Lørenskog, Norway

7. Division of Medicine and Laboratory, Institute of Clinical Medicine, Faculty of Medicine, University of Oslo, Oslo, Norway

8. Department of Technical Physics, University of Eastern Finland, Kuopio, Finland

9. Diagnostic Imaging Center, Kuopio University Hospital, Kuopio, Finland

10. School of Electrical Engineering and Computer Science, The University of Queensland, Brisbane, Australia

11. Department of Clinical and Biological Psychology, Faculty of Psychology, University of Bergen, Bergen, Norway

12. Science Service Center, Kuopio University Hospital, Kuopio, Finland

13. Division of Surgery, Institute of Clinical Medicine, Faculty of Medicine, University of Oslo, Oslo, Norway

14. Department of Cardiology, Division of Medicine, Akershus University Hospital, Lørenskog, Norway

*Corresponding author: Harald Hrubos- Strøm

Department of Otorhinolaryngology, Division of Surgery, Akershus University Hospital, Sykehusveien 25, 1478 Lørenskog, Norway

Email: harald.hrubos-strom@medisin.uio.no

Figure S1. **a**) Apnea-hypopnea index (AHI); **b**) oxygen desaturation index (ODI); **c**) desaturation duration (DesDur); **d**) desaturation severity (DesSev); **e**) log-transformed cardiac troponin I and **f**) cardiac troponin T distribution stratified by major adverse cardiovascular events (MACE).

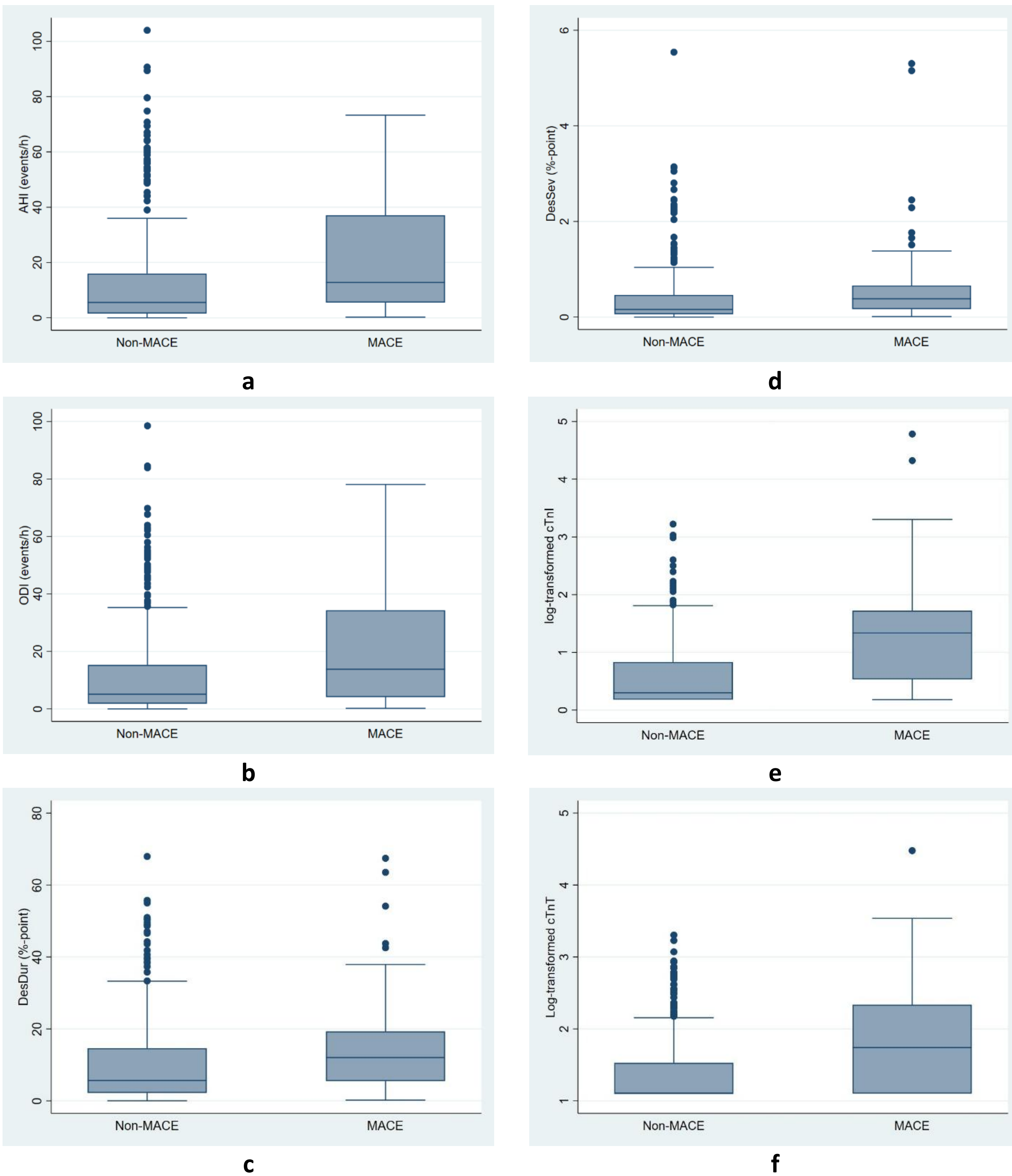

Supplement: Supplementary file 1 [file supplemental_materials_zsag013.pdf]
